# Supplementary figures and images for: A new aggressive xenograft model of human colon cancer using cancer-associated fibroblasts
Source: PeerJ. 2020 Jun 3;8:e9045. doi: 10.7717/peerj.9045 (PMC7275677; doi:10.7717/peerj.9045)

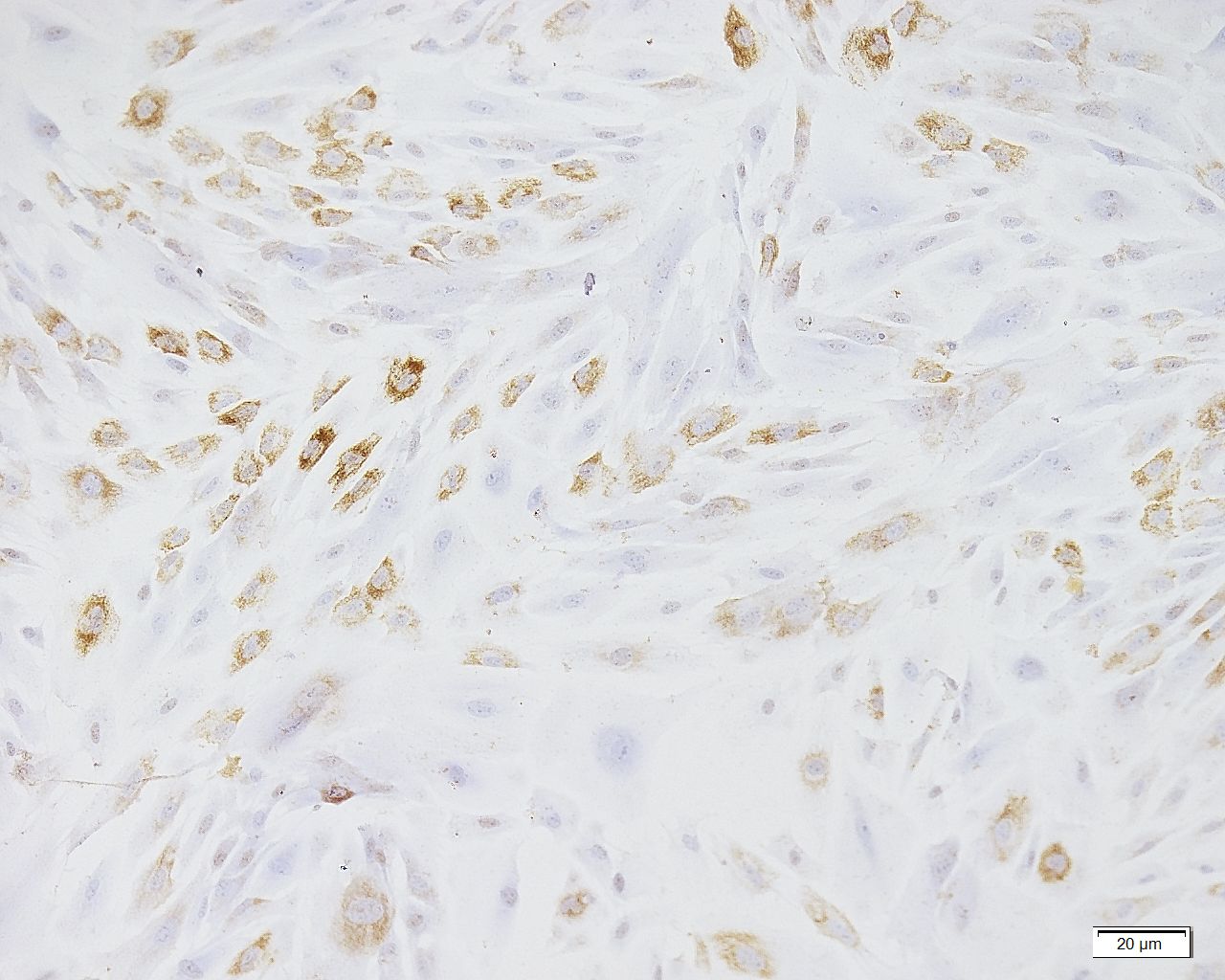

Supplement: Supplemental Information 1 — Scale bar: 20 µm. [file peerj-08-9045-s001.jpg]

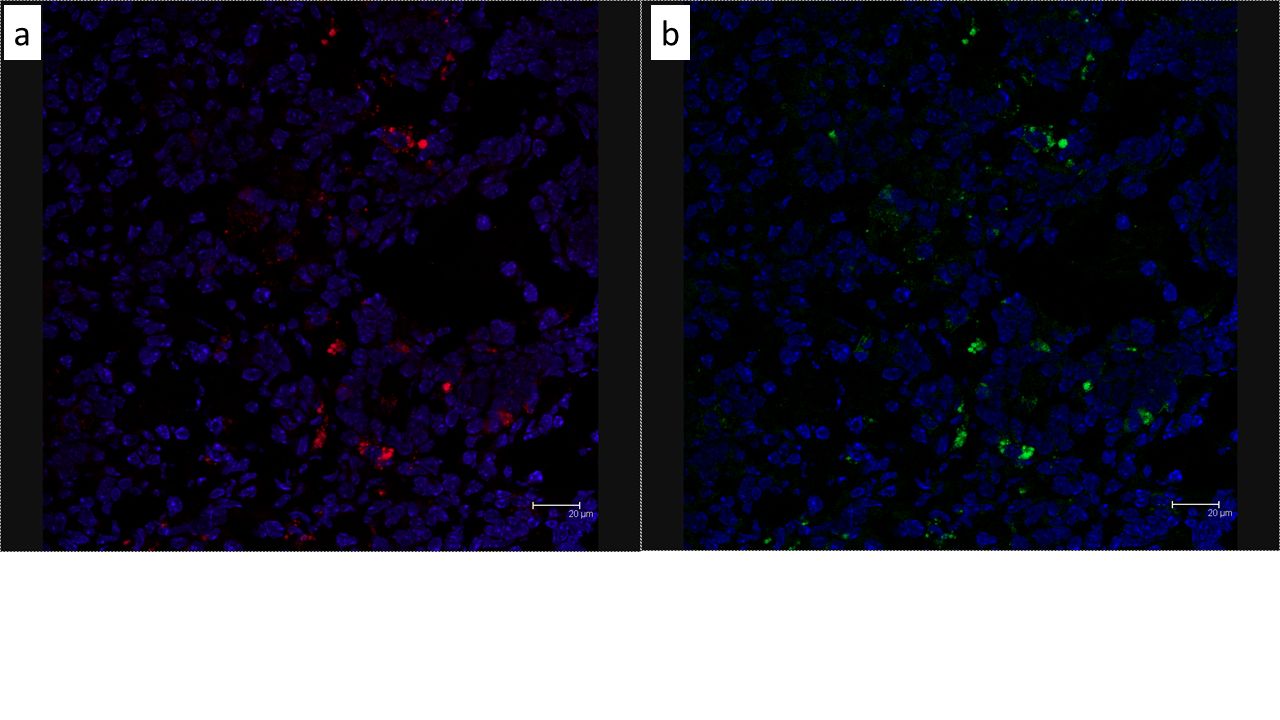

Supplement: Supplemental Information 2 — Sections were counterstained with DAPI (blue), nuclear cell marker. Original magnification, ×630. Scale bar: 20 µm. [file peerj-08-9045-s002.jpg]

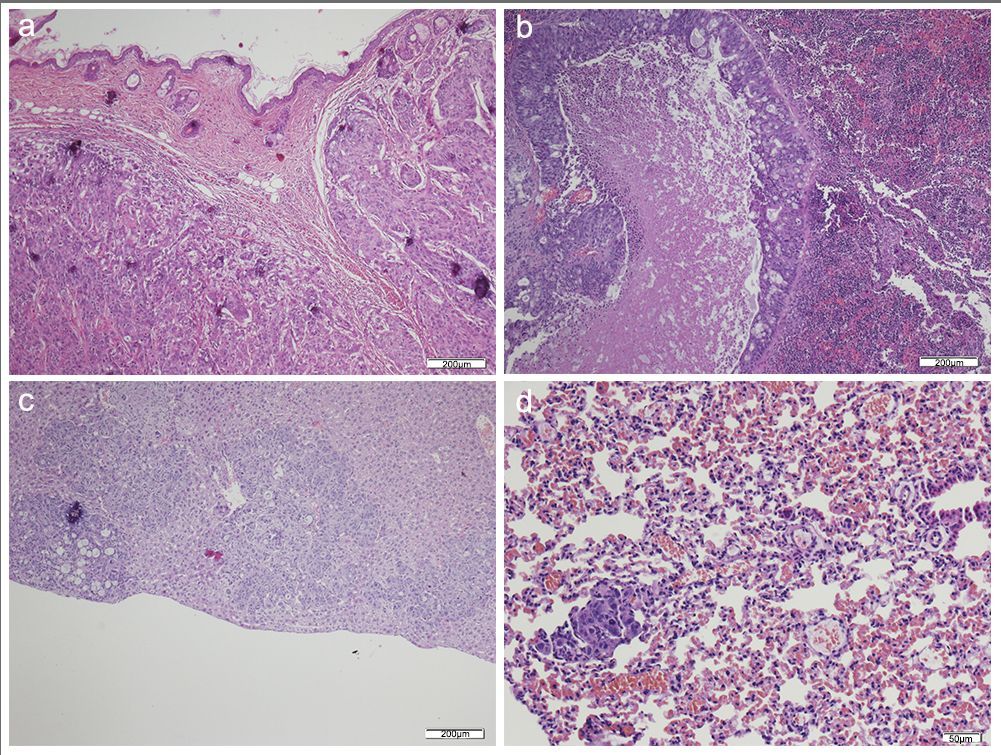

Supplement: Supplemental Information 3 — Metastasis of small and large clusters of malignant epithelial cells with desmoplastic reaction in some of them (score 3 of no. of metastasized organs) in: (A) Skin; (B) Spleen; (C) Liver; (D) Lung. (H–E, 4×). Scale bar: (A, B and C) 200 µm; (D) 50 µm. [file peerj-08-9045-s003.jpg]
