# Supplementary material for: A new aggressive xenograft model of human colon cancer using cancer-associated fibroblasts
Source: PeerJ. 2020 Jun 3;8:e9045. doi: 10.7717/peerj.9045 (PMC7275677; doi:10.7717/peerj.9045)
Supplement: Supplementary file 2 [file peerj-08-9045-s004.odt]

Supplementary Table S1. Growth of fibroblast cultures

|  | GR0UP | CASE | TIME (days) | No CELLS (Mean of two determinations) | PATIENT IDENTIFICATION |
| --- | --- | --- | --- | --- | --- |
| 1 | NF | 1 | 0 | 10000 | 34 |
| 2 | NF | 1 | 2 | 27500 | 34 |
| 3 | NF | 1 | 4 | 41250 | 34 |
| 4 | NF | 1 | 6 | 96000 | 34 |
| 5 | NF | 1 | 8 | 120000 | 34 |
| 6 | NF | 1 | 10 | 135000 | 34 |
| 7 | NF | 1 | 12 | 117500 | 34 |
| 8 | NF | 2 | 0 | 10000 | 40 |
| 9 | NF | 2 | 2 | 22500 | 40 |
| 10 | NF | 2 | 4 | 42500 | 40 |
| 11 | NF | 2 | 6 | 48750 | 40 |
| 12 | NF | 2 | 8 | 60000 | 40 |
| 13 | NF | 2 | 10 | 58750 | 40 |
| 14 | NF | 2 | 12 | 63750 | 40 |
| 15 | NF | 3 | 0 | 10000 | 49 |
| 16 | NF | 3 | 2 | 17500 | 49 |
| 17 | NF | 3 | 4 | 21250 | 49 |
| 18 | NF | 3 | 6 | 31250 | 49 |
| 19 | NF | 3 | 8 | 46250 | 49 |
| 20 | NF | 3 | 10 | 51250 | 49 |
| 21 | NF | 3 | 12 | 55000 | 49 |
| 22 | CAF | 4 | 0 | 10000 | 34 |
| 23 | CAF | 4 | 2 | 41000 | 34 |
| 24 | CAF | 4 | 4 | 121000 | 34 |
| 25 | CAF | 4 | 6 | 325000 | 34 |
| 26 | CAF | 4 | 8 | 400000 | 34 |
| 27 | CAF | 4 | 10 | 525000 | 34 |
| 28 | CAF | 4 | 12 | 340000 | 34 |
| 29 | CAF | 5 | 0 | 10000 | 40 |
| 30 | CAF | 5 | 2 | 110000 | 40 |
| 31 | CAF | 5 | 4 | 98750 | 40 |
| 32 | CAF | 5 | 6 | 126250 | 40 |
| 33 | CAF | 5 | 8 | 220000 | 40 |
| 34 | CAF | 5 | 10 | 255000 | 40 |
| 35 | CAF | 5 | 12 | 271500 | 40 |
| 36 | CAF | 6 | 0 | 10000 | 49 |
| 37 | CAF | 6 | 2 | 31125 | 49 |
| 38 | CAF | 6 | 4 | 41250 | 49 |
| 39 | CAF | 6 | 6 | 50250 | 49 |
| 40 | CAF | 6 | 8 | 106500 | 49 |
| 41 | CAF | 6 | 10 | 122500 | 49 |
| 42 | CAF | 6 | 12 | 157500 | 49 |

Twelve-day growth data of normal human fibroblasts (NF) and cancer- associated fibroblasts (CAF), obtained from three patients with sigmoid colon adenocarcinoma (identification code: 34, 40 and 49).
