# Supplementary material for: A new aggressive xenograft model of human colon cancer using cancer-associated fibroblasts
Source: PeerJ. 2020 Jun 3;8:e9045. doi: 10.7717/peerj.9045 (PMC7275677; doi:10.7717/peerj.9045)
Supplement: Supplementary file 3 [file peerj-08-9045-s005.odt]

Suplementary Table 2.  Histopathological report of HT29 transplantation in the head of the pancreas of SCID mice

| Nº | CELLS | MOUSE WEIGHT (g) | TUMOR VOLUME (mm3) | ORGAN | HISTOPATHOLOGICAL ANALYSES |
| --- | --- | --- | --- | --- | --- |
| RH1 | 1,5 millions HT29 | 19,5 | 91,61 | TUMOR | Tumor type: carcinoma Degree of differentiation: poorly differentiated Maximum size: 8.700 X 4.700 µm Desmoplasia: very slight |
| PANCREAS | 4 foci poorly differentiated infiltrating stomach submucosa |
| HEART | No evidence of tumor infiltration |
| SPLEEN | No evidence of tumor infiltration |
| SMALL INTESTINE | 1 focus well differentiated infiltrating inner muscularis tunic |
| LIVER | 3 large metastases 3 small metastases |
| LUNG | 5 small metastases |
| KIDNEY | No evidence of tumor infiltration |
| RH2 | 1,5 millions HT29 | 23,68 | 3,67 | TUMOR | Tumor type: carcinoma Degree of differentiation: poorly differentiated Maximum size: 2.421,70 X 1711,43 µm Desmoplasia: very slight |
| PANCREAS | 3 foci poorly differentiated infiltrating outer muscular stomach tunic |
| HEART | No evidence of tumor infiltration |
| SPLEEN | No evidence of tumor infiltration |
| SMALL INTESTINE | No evidence of tumor infiltration |
| LIVER | 1 small metastases |
| LUNG | No evidence of tumor infiltration |
| KIDNEY | No evidence of tumor infiltration |
| RH3 | 1,5 millions HT29 |  |  |  | Dead |
| RH4 | 1,5 millions HT29 |  |  |  | Dead |
| RH5 | 1,5 millions HT29 |  |  |  | Dead |
| RH6 | 1,5 millions HT29 |  |  |  | Dead |
| RH7 | 1,5 millions HT29 |  |  |  | Dead |
