# Supplementary material for: A new aggressive xenograft model of human colon cancer using cancer-associated fibroblasts
Source: PeerJ. 2020 Jun 3;8:e9045. doi: 10.7717/peerj.9045 (PMC7275677; doi:10.7717/peerj.9045)
Supplement: Supplementary file 4 [file peerj-08-9045-s006.odt]

Supplementary Table 3. Histopathological report of HT29 + NF transplantation in the head of the pancreas of SCID mice

| Nº | CELLS | MOUSE WEIGHT (g) | TUMOR VOLUME (mm3) | ORGAN | HISTOPATHOLOGICAL ANALYSES |
| --- | --- | --- | --- | --- | --- |
| RHFN1 | 1,5 millions HT29 + 150.000 NF | 25,13 | 65,34 | TUMOR | Tumoral type: carcinoma Degree of differentiation: moderate to poorly differentiated Maximum size: 6.533,28 X 4393,7 µm Desmoplasia: mild-moderate |
| PANCREAS | 3 foci moderate to poorly differentiated |
| STOMACH | 1 focus poorly differentiated infiltrating inner muscular stomach tunic 1 focus poorly differentiated infiltrating mucosa |
| DUODENUM | No evidence of tumor infiltration |
| SMALL INTESTINE | 1 focus poorly differentiated infiltrating mucosa |
| LARGE INTESTINE | No evidence of tumor infiltration |
| LIVER | 1 large metastases 3 medium metastases 1 small metastases |
| SPLEEN | No evidence of tumor infiltration |
| LUNG | No evidence of tumor infiltration |
| HEART | No evidence of tumor infiltration |
| KIDNEY | No evidence of tumor infiltration |
| RHFN2 | 1,5 millions HT29 + 150.000 NF | 25,8 | 239,57 | TUMOR | Tumoral type: carcinoma Degree of differentiation: moderate to poorly differentiated Maximum size: 11.045,64 X 6463,14 µm Desmoplasia: moderate |
| PANCREAS | 1 focus moderate to poorly differentiated |
| STOMACH | 1 focus poorly differentiated infiltrating outer muscular tunic |
| DUODENUM | - |
| SMALL INTESTINE | 1 focus poorly differentiated infiltrating outer muscular tunic 1 focus poorly differentiated infiltrating submucosa |
| LARGE INTESTINE | 3 foci poorly differentiated infiltrating outer muscular tunic |
| LIVER | 2 large metastases |
| SPLEEN | Multiple foci moderate to poorly differentiated infiltrating splenic capsule |
| LUNG | Multiple foci moderate to poorly differentiated |
| HEART | No evidence of tumor infiltration |
| KIDNEY | 1 focus poorly differentiated infiltrating renal capsule |
| RHFN3 | 1,5 millions HT29 + 150.000 NF | 22,43 | 28,2 | TUMOR | Tumoral type: carcinoma Degree of differentiation: mildly differentiated Maximum size: 6.545,90 X 2.882,11 µm Desmoplasia: moderate |
| PANCREAS | 1 focus poorly differentiated |
| STOMACH | 1 focus poorly differentiated infiltrating mucosa |
| DUODENUM | No evidence of tumor infiltration |
| SMALL INTESTINE | 1 focus poorly differentiated infiltrating outer muscular tunic |
| LARGE INTESTINE | No evidence of tumor infiltration |
| LIVER | 1 large metastases 1 medium metastases 2 small metastases |
| SPLEEN | No evidence of tumor infiltration |
| LUNG | No evidence of tumor infiltration |
| KIDNEY | No evidence of tumor infiltration |
| RHFN4 | 1,5 millions HT29 + 150.000 NF | 26,09 | 8 | TUMOR | Tumoral type: carcinoma Degree of differentiation: poorly differentiated Maximum size: 3.494,54 X 2.101,55 µm Desmoplasia: moderate |
| PANCREAS | 1 focus poorly differentiated |
| STOMACH | 3 foci poorly differentiated infiltrating outer muscular tunic |
| DUODENUM | No evidence of tumor infiltration |
| SMALL INTESTINE | 1 focus poorly differentiated infiltrating outer muscular tunic |
| LARGE INTESTINE | No evidence of tumor infiltration |
| LIVER | 1 medium metastases 2 small metastases |
| SPLEEN | No evidence of tumor infiltration |
| LUNG | No evidence of tumor infiltration |
| HEART | No evidence of tumor infiltration |
| KIDNEY | No evidence of tumor infiltration |
| RHFN5 | 1,5 millions HT29 + 150.000 NF |  |  |  | Dead |
