# Supplementary material for: A new aggressive xenograft model of human colon cancer using cancer-associated fibroblasts
Source: PeerJ. 2020 Jun 3;8:e9045. doi: 10.7717/peerj.9045 (PMC7275677; doi:10.7717/peerj.9045)
Supplement: Supplementary file 5 [file peerj-08-9045-s007.odt]

Suplementary Table 4. Histopathological report of HT29 + CAFs transplantation in the head of the pancreas of SCID mice

| Nº | CELLS | MOUSE WEIGHT (g) | TUMOR VOLUME (mm3) | ORGAN | HISTOPATHOLOGICAL ANALYSES |
| --- | --- | --- | --- | --- | --- |
| RHF1 | 1,5 millions HT29 + 150.000 CAFs | 25,5 | 76,86 | TUMOR | Tumor type: carcinoma Degree of differentiation: poorly differentiated Maximum size: 7.300 X 4.500 µm Desmoplasia: mild |
| PANCREAS | 1 focus poorly differentiated infiltrating stomach submucosa |
| HEART | No evidence of tumor infiltration |
| SPLEEN | No evidence of tumor infiltration |
| SMALL INTESTINE | No evidence of tumor infiltration |
| LIVER | 1 large metastases 4 medium metastases 4 small metastases |
| LUNG | 1 small metastases 1 medium metastases |
| KIDNEY | No evidence of tumor infiltration |
| RHF2 | 1,5 millions HT29 + 150.000 CAFs | 26,2 | - | TUMOR | Tumoral tissue is not recognized |
| PANCREAS | No evidence of tumor infiltration |
| HEART | - |
| SPLEEN | No evidence of tumor infiltration |
| SMALL INTESTINE | - |
| LIVER | No evidence of tumor infiltration |
| LUNG | - |
| KIDNEY | - |
| RHF3 | 1,5 millions HT29 + 150.000 CAFs | 26,2 | 563,1 | TUMOR | Tumor type: carcinoma Degree of differentiation: poorly differentiated Maximum size: 12.000 X 9.500 µm Desmoplasia: mild-moderate |
| PANCREAS | 1 focus poorly differentiated |
| HEART | No evidence of tumor infiltration |
| SPLEEN | No evidence of tumor infiltration |
| SMALL INTESTINE | Small nets poorly differenciated infiltrating serosa tunic |
| LIVER | 3 large metastases 1 medium metastases 3 small metastases |
| LUNG | 1 small metastases |
| KIDNEY | No evidence of tumor infiltration |
| RHF4 | 1,5 millions HT29 + 150.000 CAFs | 24,75 | 154,8 | TUMOR | Tumor type: carcinoma Degree of differentiation: poorly differentiated Maximum size: 9.700 X 5.542,30 µm Desmoplasia: mild |
| PANCREAS | 1 focus poorly differentiated infiltrating stomach submucosa |
| HEART | - |
| SPLEEN | No evidence of tumor infiltration |
| SMALL INTESTINE | 1 focus poorly differentiated infiltrating mucosa |
| LIVER | 2 large metastses |
| LUNG | - |
| KIDNEY | - |
| SKIN | Multiple foci of poorly differentiated carcinoma infiltrating: dermal and hypodermal skin and panniculus carnosus |
| RHF5 | 1,5 millions HT29 + 150.000 CAFs | 27 | 93,73 | TUMOR | Tumoral type: carcinoma Degree of differentiation: poorly differentiated Maximum size: 6.778,11 X 5.166,35 µm Desmoplasia: mild-moderate |
| PANCREAS | 1 focus poorly differentiated infiltrating stomach mucosa |
| HEART | No evidence of tumor infiltration |
| SPLEEN | No evidence of tumor infiltration |
| SMALL INTESTINE | No evidence of tumor infiltration |
| LIVER | 1 large metastases 1 medium metastases 9 small metastases |
| LUNG | 4 small metastases |
| KIDNEY | No evidence of tumor infiltration |
| RHF6 | 1,5 millions HT29 + 150.000 CAFs | 25,8 | 169,88 | TUMOR | Tumor type: carcinoma Degree of differentiation: poorly differentiated Maximum size: 10.800 X 5.500 µm Desmoplasia: mild-moderate |
| PANCREAS | 2 foci poorly differentiated infiltrating stomach submucosa |
| HEART | - |
| SPLEEN | 4 foci poorly differentiated |
| SMALL INTESTINE | 1 focus poorly differentiated infiltrating submucosa |
| LIVER | 2 large metastases 1 medium metastases 2 small metastases |
| LUNG | - |
| KIDNEY | - |
| RHF7 | 1,5 millions HT29 + 150.000 CAFs |  |  |  | Dead |
| RHF8 | 1,5 millions HT29 + 150.000 CAFs |  |  |  | Dead |
| RHF9 | 1,5 millions HT29 + 150.000 CAFs | 23,06 | 207,03 | TUMOR | Tumoral type: carcinoma Degree of differentiation: moderate to poorly differentiated Maximum size: 6.825,90 X 8.560,89 µm Desmoplasia: moderate |
| PANCREAS | 2 foci modertate to poorly differentiated |
| STOMACH | 3 foci poorly differentiated infiltrating outer muscular stomach tunics 2 foci poorly differentiated infiltrating  submucosa |
| DUODENUM | No evidence of tumor infiltration |
| SMALL INTESTINE | No evidence of tumor infiltration |
| LARGE INTESTINE | No evidence of tumor infiltration |
| LIVER | 3 large metastases |
| SPLEEN | No evidence of tumor infiltration |
| LUNG | No evidence of tumor infiltration |
| HEART | No evidence of tumor infiltration |
| KIDNEY | No evidence of tumor infiltration |
| RHF10 | 1,5 millions HT29 + 150.000 CAFs | 25,01 | 143,9 | TUMOR | Tumoral type: carcinoma Degree of differentiation: poorly differentiated Maximum size: 9.674,22 X 5.358,74 µm Desmoplasia: mild-moderate |
| PANCREAS | 1 focus poorly differentiated |
| STOMACH | 1 focus poorly differentiated infiltrating  submucosa |
| DUODENUM | No evidence of tumor infiltration |
| SMALL INTESTINE | 1 focus poorly differentiated infiltrating mucosa 1 focus poorly differentiated infiltrating  outer muscular tunics |
| LARGE INTESTINE | No evidence of tumor infiltration |
| LIVER | 1 large metastases 1 medium metastases 4 small metastases |
| SPLEEN | No evidence of tumor infiltration |
| LUNG | 7 small foci poorly differentiated |
| HEART | No evidence of tumor infiltration |
| KIDNEY | No evidence of tumor infiltration |
